# Hyperglycaemia in the Newborn Infant. Physiology Verses Pathology

**DOI:** 10.3389/fped.2021.641306

**Published:** 2021-07-21

**Authors:** Kathryn Beardsall

**Affiliations:** ^1^Department of Paediatrics, University of Cambridge, Cambridge University Hospitals National Health Service Foundation Trust, Cambridge, United Kingdom; ^2^Neonatal Unit, Cambridge University Hospitals National Health Service Foundation Trust, Cambridge, United Kingdom

**Keywords:** hypoxic ischaemia, hyperglycaemia, preterm, glucose, hypoglycaemia, monitoring

## Abstract

Hyperglycemia is common in newborns requiring intensive care, particularly in preterm infants, in sepsis and following perinatal hypoxia. The clinical significance, and optimal intervention strategy varies with context, but hyperglycaemia is associated with increased mortality and morbidity. The limited evidence for optimal clinical targets mean controversy remains regarding thresholds for intervention, and management strategies. The first consideration in the management of hyperglycaemia must be to ascertain potentially treatable causes. Calculation of the glucose infusion rate (GIR) to insure this is not excessive, is critical but the use of insulin is often helpful in the extremely preterm infant, but is associated with an increased risk of hypoglycaemia. The use of continuous glucose monitoring (CGM) has recently been demonstrated to be helpful in targeting glucose control, and reducing the risk from hypoglycaemia in the preterm infant. Its use in other at risk infants remains to be explored, and further studies are needed to provide a better understanding of the optimal glucose targets for different clinical conditions. In the future the combination of CGM and advances in computer algorithms, to provide intelligent closed loop systems, could allow a safer and more personalized approached to management.

## Introduction

Although *in utero* glucose levels are normally maintained between 4 and 6 mmol/l hyperglycaemia is common in newborns requiring intensive care, particularly in preterm infants, in sepsis and following perinatal hypoxia (1, 2). Transient hyperglycaemia may be a physiological response to stress but when prolonged is associated with significant morbidity and mortality. Hyperglycaemia has variably been defined based on absolute thresholds, as well as length of exposure and association with glycosuria. Threshold definitions range from >7 to >13.3 mmol/l (>126–239 mg/dl) (3–6). The most common definition is blood glucose (BG) >10 mmol/l (180 mg/dl) (3). However, the European Society for Paediatric Gastroenterology Hepatology and Nutrition (ESPGHAN) recommends avoiding glucose levels >8 mmol/l (145 mg/dl), because they are associated with increasing morbidity and mortality (7). Hyperglycaemia is most commonly seen in the extremely preterm infant in the first week of life, reports varying between 20 and 86% (1, 8–17). However, glycaemic instability and hyperglycaemia remain in these infants even at the time of discharge (1, 8–16). Hyperglycaemia is also prevalent in infants following hypoxic ischaemic (HI) insults (18), associated with sepsis and in neonatal diabetes which has recently been reviewed (19). The use of systemic steroids, inotropes, and caffeine (20, 21), as well as stress associated with intubations can also increase glucose levels (1).

The limited evidence for optimal clinical targets mean controversy remains regarding thresholds for intervention and management strategies. The first consideration in the management of hyperglycaemia must be to ascertain potentially treatable causes. Hyperglycaemia in the newborn may be initially considered an acute catabolic response to stress, but prolonged hyperglycaemia is associated with a poor prognosis. There are numerous studies reporting its association with increased morbidity and mortality, and there are biologically plausible causal pathways. These include the direct effects of hyperglycaemia *per se*, as well as the effect of relative insulin deficiency. As hyperglycaemia is an easily modifiable risk factor for poor outcomes it is important to understand potential mechanisms of harm, and intervention strategies that could improve outcomes.

## Hyperglycaemia in the Preterm Infant

The prevalence of hyperglycaemia is inversely related to gestational age with extremely preterm infants at most risk. Many preterm morbidities as well as mortality are associated with increased hyperglycaemic exposure (22). These include retinopathy of prematurity (23), chronic lung disease (24), necrotizing enterocolitis (NEC) (25), hypernatraemia, and reduced white matter in the brain at term (26). Associations are often reduced or lost after adjusting for gestational age and birth weight, as there is a close relationship between hyperglycaemia and immaturity. It is similarly difficult to separate how much hyperglycaemia is a marker of the metabolic disturbance, that is the primary etiology for poor outcome, as opposed to contributing itself to that causal pathway. However, even after adjustment for gestational age, early hyperglycaemia has been shown to be associated with an increased risk of death or sepsis, OR 5.07 (95% CI 1.06–24.3) (27). Furthermore, hyperglycaemia has been associated with poor growth up to 2 years of age (28, 29). The implications of hyperglycaemia and prolonged catabolism on longer term metabolic and neurocognitive outcomes for preterm infants remains to be determined.

### Pathogenesis of Hyperglycaemia in the Preterm Infant

In preterm infants hyperglycaemia can be considered to result from a combination of excess glucose delivery, counter regulatory response to stress and infection, and the impact of prematurity and growth restriction on insulin secretion and sensitivity (12, 30, 31).

#### Central and Peripheral Glucose Insensitivity and Insulin Resistance

In the healthy adult glucose infusions (6 mg/kg/min) completely suppress endogenous glucose production. However, in the preterm neonate glucose production is not suppressed in the same way by glucose infusions. Studies in the newborn have shown glucose levels can reach >13.9 mmol/l (250 mg/dl), or glucose infusion rates (GIRs) >16 mg/kg/min before glucose production is suppressed (32–34). Similarly large reductions in the GIRs may not alter glucose production rates, when one might anticipate it would lead to an increase in gluconeogenesis (35). Persistent endogenous glucose production, in spite of glucose infusion, has been shown in preterm infants even at the age 2–5 weeks (36). This may in part be due to immature expression of glucose transporters (GLUT), particularly glucose transporter 2 (GLUT2) and glucose transporter 4 (GLUT4). Low GLUT 2 levels in the liver may lead to lack of glucose sensitivity, and continued hepatic glucose production (37). The less abundant insulin sensitive tissues (adipose and skeletal muscle), and low GLUT4 expression in muscle, may also result in reduced insulin mediated glucose uptake in preterm infants (2, 38). Increased levels of pro-inflammatory cytokines (tumor necrosis factor-α, interleukin-1, interleukin-6), secondary to chorioamnionitis, sepsis or NEC, may also lead to insulin resistance, and altered insulin receptor signaling. Intensive care interventions, such as the use of inotropes and corticosteroids, also increase insulin resistance and suppress insulin secretion.

#### Relative Insulin Deficiency

*In utero* studies suggest that insulin levels increase toward term, and immaturity of the β-cells may result in insufficient insulin secretion (6). GLUT 2 transporters are involved in glucose stimulated insulin secretion from the pancreas, but fetal pancreatic β cells do not express GLUT 2 until 7 months (39, 40), impacting on the β cell's response to hyperglycaemia (32). In the preterm infant the insulin secretory response to glucose is reduced compared with the term infant, but increases postnatally over a number of weeks (41). Preterm infants are often also growth restricted, and this is associated with reduced β cell mass (42, 43). However, these changes are dependent on the model and timing of growth restriction (44, 45). The levels of proinsulin (a less active precursor to insulin), are high in preterm neonates, suggesting that the processing of proinsulin in β-cells is partially defective. This relative insulin deficiency may contribute to reduced insulin like growth factor 1 (IGF-I) generation, with further impacts on metabolism and growth.

#### Feeding and Incretins

Incretins play an important role in augmenting insulin secretion in adults (46, 47), and the delay in enteral feeding of preterm infants means the normal stimulation of incretins does not occur (48). In the preterm infant glucose control often improves once enteral feeds have been established, but even when enteral feeds are given, preterm infants do not demonstrate an equivalent incretin response compared to that seen in term infants (49).

### Clinical Consequences of Hyperglycaemia

An initial counter regulatory response and transient hyperglycaemia may be beneficial in acute stress, and may be considered physiological. However, prolonged hyperglycaemia in critical illness has been associated with poor prognosis. Furthermore, for the preterm infant, the period from birth to term is a critical period of development, and even shorter periods of hyperglycaemia may be harmful. It remains unclear as to whether harmful effects of dysglycaemia are mediated by the primary effects of hyperglycaemia per se, or the effects of relative insulin deficiency, with both potentially having short and long term clinical consequences.

#### Primary Role of Hyperglycaemia

Hyperglycaemia is harmful to cells and can lead to an over expression of insulin independent glucose transporters (GLUT-1, GLUT-2, and GLUT 3), which leads to an increase in glucose uptake by endothelial, hepatic, immune, and nerve cells (50). Glucose overload can cause an increased generation of oxygen free radicals, which can cause mitochondrial dysfunction and increased apoptosis. Hyperglycaemia also impairs leukocyte phagocytic function, decreases complement function, increases pro-inflammatory cytokines, and impairs neutrophil chemotaxis, all leading to an increased susceptibility to infections (51–54).

Independent manipulation of BG and insulin levels in an animal model of hyperglycaemia (burn injured parenterally fed rabbit), demonstrated survival to be better in the normoglycaemic groups (89% verses those with hyperglycaemia 53–64%) (55). Recent data have also shown a causal pathway linking hyperglycaemia to an increased risk of microbial gut translocation in both animal models and adult studies (56). Persistent hyperglycaemia has been associated with NEC, OR 9.49 (95% CI 1.52–59.3) and infection, OR 3.79 (1.40–10.20) (1, 25, 27) which are two major causes of mortality and morbidity for preterm infants (57, 58).

Hyperglycaemia can lead to an osmotic diuresis, hypernatraemia, and electrolyte imbalance and has been associated with intraventricular hemorrhage (IVH) (25, 59, 60). More significant may be the impact of hyperglycaemic on nervous system development and injury in animal models (61). Hyperglycaemia increases central nervous system permeability, oxidative stress, and leads to microglia activation and astrocytosis, as well as regulation of DNA repair mechanisms, compromising neuronal and glial cell integrity (62). This can lead to long-term changes in synaptogenesis and behavior (63). Clinical correlates include the finding in a cohort of extremely preterm infants that hyperglycaemia >8.3 mmol/L(150 mg/dl) on the first day of life was an independent risk factor for white matter reduction on term MRI (26). Increased BG concentrations have also been associated with decreased total absolute band power on EEG, a measure expressing background brain activity, and associated with long term outcomes (64). A large retrospective study, (including 443 preterm infants) showed hyperglycaemia to be associated with lower survival without neurodevelopmental disability at 2 years of age, but this did not remain significant after adjusting for gestational age, birth weight z-score, and socioeconomic status (65). However, the close relationship between hyperglycaemia and gestational age make separation of the causal effect of hyperglycaemia from that of immaturity challenging. Data in press from the Swedish EXPRESS cohort shows that hyperglycaemia is associated with worse motor outcomes in early childhood (after multivariate adjustment). Further long term follow up studies are required to explore the long term impact of hyperglycemia per se, and different management strategies.

#### Impact of Relative Insulin Deficiency

Hyperglycaemia may also be considered a marker of relative insulin deficiency, and this may have independent effects to those of hyperglycaemia. In both animal and human models insulin has been shown to improve innate immunity, and to suppress pro-inflammatory products, whilst increasing anti-inflammatory cytokines (66–70). Insulin deficiency may be associated with reduced expression of nitric oxide synthase (iNOS), and insulin may be protective by prevention of excess nitric oxide release. Insulin can also improve cardiac function (55), and in patients post myocardial infarction and in sepsis, the combination of glucose and insulin infusion improves cardiac function (71). Insulin infusions can reduce proteolysis, and in burns have a positive impact on protein synthesis and wound healing (72–74). Relative insulin deficiency can also lead to low IGF-I levels, which can be detrimental, as IGF-I is an important mediator of growth in the neonatal period. Starvation and critical illness lead to suppression of IGF-I levels, and IGF-I administration has been shown to increase nitrogen balance in catabolic states (75, 76). IGF-I is also an important growth factor influencing perinatal pancreatic development, with low levels leading to increased apoptosis, and potentially resulting in reduced β-cell mass. Therefore, insulin deficiency has implications in the preterm infants for growth, as well as longer term metabolic health.

### Clinical Interventions for the Management of Hyperglycaemia

Thresholds for intervention remain controversial, but the recent ESPGHAN and ASPEN guidelines clearly advise avoiding glucose levels >8 mmol/l (145 mg/dl) (7), or >8.3 mmol/l (150 mg/dl) (77, 77). Approaches to management should always involve reviewing the context of hyperglycaemia, with particular consideration as to whether there is evidence for acute illness, such as infection which requires treatment. Limitation of excess glucose intake should then be considered, and insulin used in the context of wishing to maintain postnatal growth when appropriate (4). Simply increasing calorie intake may be detrimental (14), but optimizing amino acid intakes and the use of insulin has the theoretical potential to improve lean body mass, and pancreatic function (78). The potential benefits of insulin however need to be balanced with the risks of hypoglycaemia, and therefore careful monitoring of glucose levels should be undertaken on any infant on insulin.

#### The Importance of Parenteral Nutrition

The relationship between glucose infusions and hyperglycaemia is not consistent. Some studies have shown a direct positive relationship between GIRs and risk of hyperglycamia, other studies have not found such a clear relationship (1, 20, 60, 79, 80). Differences may relate to the rates of glucose being infused, with excess rates clearly associated with hyperglycaemia, but the impact of lower rates of glucose infusion more nuanced. At lower GIRs the influence of differences in other components of parenteral nutrition (PN) may play an important role. Amino acids stimulate insulin secretion, and low plasma arginine levels have been associated with hyperglycaemia (81). Neonates receiving amino acid infusions in addition to glucose have higher insulin levels (82). A Cochrane meta-analysis concluded that higher amino acid intake in PN was associated with a reduction in hyperglycaemia. Therefore, reducing PN al intake may be counterproductive, if it reduces amino acid intake along with glucose load. One study that reported on the impact of a change in clinical practice, aimed at limiting dextrose intake (to minimum of 4 mg/kg/min), demonstrated a reduction in the prevalence of hyperglycaemia, use of insulin and mortality. However, total protein and energy intakes were also higher after the intervention, which cannot therefore be viewed as a simple intervention on dextrose intake (83). Lipid infusions may have a beneficial effect, by reducing the glucose load whilst maintaining energy intakes, they can reduce the prevalence of hyperglycaemia (84). However, in excess or in acute illness, lipids have been reported to contribute to hyperglycaemia (85).

A single center study in Norway showed implementation of an enhanced PN protocol was associated with an increased prevalence of severe hyperglycaemia, and higher mortality (14). However, in the multivariate analysis, the enhanced PN regimen per se was not predictive of mortality, it was the early severe hyperglycaemia that was the strongest risk factor for death. After adjusting for potential confounding variables, early severe hyperglycaemia was an independent risk factor for death (OR, 4.68; 95% CI, 1.82–12.03), greater than that of gestational age (odds ratio, 0.62; 95% CI, 0.49–0.79). Attempts to reduce the prevalence of hyperglycaemia, by controlling glucose intake, have included the use of continuous glucose monitoring (CGM) (86). In this study, glucose delivery was determined by a computer guided GIR that was supported by either real time CGM (intervention), or intermittent BG levels (control). Those in the intervention arm (using CGM), showed an increased median time in target (72–144 mg/dL, 4–8 mmol/l), of 84% compared to 68% in controls.

There are good reasons to ensure that excess glucose delivery is avoided, as exceeding maximum glucose oxidation rates can cause increased carbon dioxide production, lipogenesis and fat deposition including liver steatosis (87). High rates of glucose infusion and hyperglycaemia can themselves lead to increased insulin resistance and endogenous glucose production (88). In appropriately grown preterm newborns the maximum rate of glucose oxidation has been estimated to be 6–8 mg/kg/min, compared to term infants, and infants on long term PN where maximum glucose oxidation rates are 12 mg/kg/min (89). When determining glucose requirements, and optimal management for hyperglycaemia it is important to consider the metabolic phase of illness. During the acute phase of critical illness, such as sepsis, increasing glucose and nutritional intake will not promote anabolism and may be detrimental (90). In contrast, in a more stable preterm infant, where growth and anabolism are the priority, the approach to hyperglycaemia would normally be to favor optimizing nutritional delivery. ESPGHAN recommend parenteral glucose intake of 4–8 mg/kg/min on day 1 (and during any subsequent acute illness such as infection), rising to 8–10 mg/kg/min over the subsequent 2–3 days to allow for growth (7). Both ESPGHAN and the American Society for Parenteral and Enteral Nutrition recommends maintaining GIRs (<12 mg/kg/min), but not reducing to <4 mg/kg/min (77). If hyperglycaemia persists (>10 mmol/l, 180 mg/dL), it is then recommended that insulin treatment should be started (7).

#### The Role of Insulin

A number of small single center studies suggest that the use of insulin can help to maintain nutritional intake. These studies showed that infants who were hyperglycaemic, and randomized to treatment with insulin, tolerated higher GIRs, and had greater weight gain, in comparison to those treated with reduced glucose intake, who remained catabolic for longer (91–97). These findings may be related to a decrease in proteolysis, but also protein synthesis. One small study raised concern that insulin infusions significantly increased lactic acidosis, and did not impact on protein synthesis. However, this study infused high rates of glucose (14–17 mg/kg/min) without the infusion of any amino acids (98).

There are limited data from larger interventional studies in the preterm newborn. The NIRTURE Trial, a large multicentre randomized controlled trial used early insulin treatment prior to the onset of hyperglycaemia, with the aim of promoting anabolism. The trial did not demonstrate benefits and was stopped early on the grounds of futility. The study was important though in highlighting the high prevalence of clinically “silent” hypoglycaemia in both study arms. These data were achieved by uniquely collecting data on glycaemic exposure using CGM (blinded to the clinical team) and raised concerns about the challenges of insulin treatment (13).

The use of insulin to achieve “tight” glucose control has been widely debated since the landmark paper of van den Berghe which showed dramatic improvements in adult intensive care outcomes in patients randomized to tight glucose control (99). Many studies trying to replicate the positive findings of this study have raised concerns about, or been stopped early, due to the risk of severe hypoglycamiaemia (100, 101). The largest adult study showed increased risk of death in the intensive study arm (OR 1.14; 95% CI 1.02–1.28; *P* = 0.02), but highlighted the association of hypoglycaemia with mortality (102). In this context tight glucose control refers to glucose levels being maintained within a much narrower “normoglycaemic” range (typically 4–6 mmol/l), than standard care which aims to prevent hyperglycaemia (typically >8–10 mmol/l). Further differences between studies have been highlighted including: underlying reason for patients requiring intensive care, ability to achieve target levels of control and the early use of PN (103, 104). The use of PN having been shown, more recently, to be harmful, in adult and PICU. Preplanned analyses in these studies showing harmful effects being related to aminoacids, but not glucose or lipids (105). There are important differences in the preterm infant in NICU, compared with the adult in ITU, in relation to the importance of growth on survival, and differences in prevention of hyperglycaemia compared to tight glucose control.

The HINT trial is the only trial to explore tight glycaemic control in preterm infants. In this study the intervention arm “tight control” targeted glucose levels of 4–6 mmol/L (72–108 mg/dL), compared to the unit standard of care which was 8–10 mmol/L (144–180 mg/dL). The study showed high rates of hypoglycaemia in both study arms and variable effects on growth parameters (106). The study reported no overall effect on survival without neurodevelopmental delay, intelligence scores or motor skills at seven years of age, although there was beneficial effect in those who actually reached the target of 4–6 mmol/L (72–108 g/dL), but power was limited for assessing such outcomes. The effects of hypoglycaemia also had the potential for masking any effects of prevention of hyperglycaemia. More recently a large study from the National Swedish EXPRESS Cohort demonstrated, insulin treatment of hyperglycaemia in the first 28 days of life, was associated with lower 28- and 70-day mortality (17). However, in this retrospective study there were no clear criteria either for starting or modifying insulin therapy, or fixed glucose target within the different study sites.

Challenges in insulin treatment in preterm babies relate to the combination of rapidly changing insulin sensitivity, the difficulty of consistent insulin delivery, and the low frequency of glucose monitoring. Hyperglycaemia itself causes insulin resistance and following increasing insulin to regain normoglycaemia, insulin requirements often fall, and this increases the risk of hypoglycaemia (13). Insulin is easily adsorbed onto intravenous lines, and the use of large volume syringes for delivery at small infusion rates makes insulin delivery unpredictable (107, 108). Monitoring of glucose levels in preterm infants is often infrequent, and studies using CGM have shown that real time CGM alone, or in combination with computer algorithms, has the potential to reduce the prevalence of hyperglycaemia without increasing the risk of hypoglycaemia (91, 109). Furthermore, a recent international multicentre trial has demonstrated that the use of CGM in preterm infants can safely support the targeting of glucose control without causing hypoglycaemia, and is cost effective (110, 111). However, optimal target glucose levels remain to be determined.

### Hypoxic Ischaemic Encephalopathy

Both hyperglycaemia and hypoglycaemia are common in babies following perinatal HI insult. The etiology of hyperglycaemia following HI, in comparison with that of the preterm infant, is predominantly driven by the effects of stress hormones and tissue damage from hypoxia. Although hypoglycaemia has traditionally been considered a more significant risk, there is increasing evidence that hyperglycaemia is a modifiable mediator of long-term morbidity (18). Hyperglycaemia is reported in 50% of babies using intermittent BG testing, and CGM has revealed that exposure to hyperglycaemia is often more frequent and prolonged (112, 113). Pediatric intensive care studies have also shown longer duration, higher peak glucose levels, and increased glucose variability are all associated with mortality and morbidity (114).

In the analyses of the cool cap study, a multicenter trial of cooling for HIE, hyperglycaemia was confirmed as an independent risk factor for poor outcomes at 18 months (18). Further *post-hoc* analyses, after adjusting for Sarnat stage and 5 min Apgar score, only hyperglycaemic infants randomized to hypothermia had reduced risk of death and/or severe neurodevelopmental disability at 18 months (adjusted risk ratio: 0.80, 95% CI 0.66–0.99). This suggests that early glycaemic profile in infants with moderate-to-severe HIE identifies those at most risk of multi-organ dysfunction and most likely to benefit from therapeutic hypothermia (115). In neonates with encephalopathy, even after adjusting for hypoxia-ischemia severity, epochs of hyperglycaemia were associated with worse neural injury, as well as global brain function and seizures (116, 117). Whether hyperglycaemia causes neuronal injury or is simply a marker of severe brain injury is yet to be determined (116, 117).

Many potential causal mechanisms have been implicated in infants with HIE: dyslipidemia, inflammatory cytokine production, endothelial dysfunction, hypercoagulation, glucose toxicity, increased cellular apoptosis, and over-production of superoxide. However, there are potential differences in impact related to maturity of the newborn nervous system compared to similar ischaemic injuries in adults (118). Deleterious effects on the nervous system may be related to increased hyperglycaemia-induced blood-brain barrier permeability, oxidative stress, and microglia activation, which compromise neuronal and glial cell integrity (62, 119). However, optimal glucose targets for infants following HI encephalopathy remain to be determined.

## Conclusion

Hyperglycaemia is common in newborns requiring intensive care, particularly in preterm infants, and following perinatal hypoxia. The pathogenesis and clinical significance varies in each context, but hyperglycaemia is associated with increased mortality and morbidity. The limited evidence for optimal targets that impact on long term outcomes mean controversy remains regarding thresholds for intervention, and management strategies. The optimal glucose targets for infants during the acute phase of critical illness are likely to differ from those in a more stable state, when trying to achieve growth and anabolism. The first consideration in the management of hyperglycaemia must be to ascertain potentially treatable causes, followed by calculation of the GIR, to ensure it is not excessive. In term infants who are acutely unwell, restricting GIRs is likely to be more appropriate, whereas in stable extremely preterm infants where growth is considered a primary objective, one might prioritize nutritional intake with addition of insulin (4). Optimal target glucose levels remain to be determined but real-time glucose measurements and innovations in metabolomics will provide a better understanding of pathological mechanisms. This understanding, combined with real time CGM and advances in computer algorithms to provide intelligent closed loop systems, should allow a safer and more personalized approached to management in the future.

## Author Contributions

The author confirms being the sole contributor of this work and has approved it for publication.

## Conflict of Interest

The author declares that the research was conducted in the absence of any commercial or financial relationships that could be construed as a potential conflict of interest.
